# Men under the age of 55 years with screen detected prostate cancer do not have less significant disease compared to older men in a population of patients in Australia

**DOI:** 10.1186/s12894-015-0117-3

**Published:** 2015-12-30

**Authors:** Nandu D. Dantanarayana, Tania Hossack, Paul Cozzi, Andrew Brooks, Howard Lau, Warick Delprado, Manish I. Patel

**Affiliations:** University of Sydney, Discipline of Surgery, Sydney, Australia; Department of Urology, Westmead Hospital, Sydney, Australia; St George Hospital, Sydney, Australia; Douglass Hanley Moir Pathology, Sydney, Australia

**Keywords:** Insignificant, Prostate Cancer, PSA testing, Prostatectomy, Biopsy

## Abstract

**Background:**

The American Urological Association (AUA) changed their Prostate-Specific Antigen (PSA) screening guidelines in 2013 to not recommend testing in men under 55 years of age without significant risk factors (such as a family history of prostate cancer or African ethnicity). The AUA argues that the rates of 'insignificant' prostate cancer (PC) in men under 55 are so high that the potential harms of PSA-testing in this population (over diagnosis and overtreatment) outweigh the benefits (early detection and treatment). Our study aims to identify and compare the rates of insignificant and high-risk PC in men diagnosed with PC ≤55 years and >55 years in two centres in Sydney, Australia.

**Methods:**

Men with an abnormal screening PSA or DRE and diagnosed with PC by prostate biopsy were included in this study. A consecutive series of men were accrued from two major urology centres between the years 2006 and 2014. The analysis was divided into two parts, the first compared PC biopsy characteristics between men aged ≤55 years and those >55 years. The second analysis compared the prostatectomy pathological characteristics between the two groups. Differences were analysed by Chi squared and significance set at *p* < 0.05.

**Results:**

A total of 598 prostate biopsies and 723 prostatectomy matched subjects were included. On prostate biopsies, 14.0 % of men ≤55 years and 11.9 % of men >55 years had insignificant PC (X^2^ = 0.32, df = 1, *p* = 0.57), whilst 24.7 % of men ≤55 years and 25.1 % of men >55 years had high-risk PC (X^2^ = 0.007, df = 1, *p* = 0.93). On prostatectomy specimens, 9.1 % of men ≤55 years and 6.5 % of men >55 years had insignificant PC (X^2^ = 1.25, df = 1, *p* = 0.26), whilst 20.0 % of men ≤55 years and 24.0 % of men >55 years had high-risk PC (X^2^ = 0.83, df = 1, *p* = 0.36).

**Conclusion:**

We found no significant difference in the rates of insignificant and high-risk PC between men ≤55 years and >55 years, in either the prostate biopsies or prostatectomy specimens. Further trials need to be performed with comparable sample sizes and controlling of risk factors to assess the utility of PSA screening in younger men.

## Background

Prostate Specific Antigen (PSA) testing remains controversial with a diverse range of recommendations on whom to offer testing to amongst authorities worldwide [1]. In 2013, the American Urological Association (AUA) changed their PSA testing guidelines to not recommend testing in men under 55 years of age without significant risk factors (such as a family history of prostate cancer or African ethnicity) [2]. This is in contrast to the 2009 AUA guidelines that recommended PSA screening start at 40 years. The AUA argues that the rates of ‘insignificant’ prostate cancer in men under 55 are so high that the potential harms of PSA-testing in this population (over diagnosis and overtreatment) outweigh the benefits (early detection and treatment). The concept of insignificant prostate cancer is evolving but generally refers to a low grade, small volume, organ confined prostate cancer that is unlikely to manifest clinically in a patient’s lifetime without treatment [3].

Furthermore, the AUA cites a lack of high-quality evidence to support PSA screening in men 40–55 years. Specifically, two recently performed large randomized clinical trials– the Prostate, Lung, Colorectal and Ovarian Cancer screening trial (PLCO) and European Randomised Study of Screening for Prostate Cancer (ERSPC) did not include men under 55 years and therefore do not inform the decision [4, 5].

In light of the AUA guideline changes, we aim to identify the rates of insignificant and high-risk prostate cancer in men diagnosed with prostate cancer in two centres in Sydney, Australia. In contrast to the AUA, we hypothesise that there is no significant difference between the rates of insignificant and high-risk cancer between men ≤55 years and >55 years.

## Methods

Between January 2006 and February 2014 men with an abnormal screening PSA or DRE subsequently diagnosed with PC by prostate needle biopsy were included in this study. A consecutive series of patients were derived from three surgeons at Westmead hospital (Sydney, Australia) and one surgeon at St George hospital (Sydney, Australia). Patient data was de-identified and hence individual patient consent was not required for inclusion in the study. Data collected included patient age, pre-biopsy PSA score and clinical staging - obtained from audit databases maintained by each urologist in the study. Prostate biopsy data (age at biopsy, Gleason score, number of positive cores, tumour volume, PSA density) and radical prostatectomy data (age at prostatectomy, Gleason score, tumour volume, pathological staging) was obtained via histopathology reports stored on electronic databases at Douglass Hanley Moir (DHM) – a pathology company, all four surgeons use exclusively for reporting. African-American/Australian-African patients were excluded from the study since this is a known prostate cancer risk factor.

At DHM, radical prostatectomy specimens are fully processed but not whole mounted. They are embedded using standard cassettes, with each slide of the prostate fully embedded as quadrants in four separate cassettes. As such, all of the tissue is processed and examined. All of the tumour areas are drawn on a schematic and the diagram is used to calculate tumour volume, Gleason score and presence of extraprostatic extension.

The initial analysis compared PC biopsy characteristics between men ≤55 years and those >55 years at time of biopsy. From the prostate biopsy data, subjects were identified as having insignificant prostate cancer as per the Epstein criteria [6] (PSA density <0.15 ng/ml per gram, no Gleason pattern 4 / 5, <3 positives cores and <50 % involvement per core) or high-risk prostate cancer as per the D’Amico risk grouping [7] (at least one of Gleason score >7, PSA >20, Clinical stage > T2b). Whilst the Epstein biopsy criteria is controversial – with some advocating modifications such as including the presence of perineural invasion to increase criteria accuracy - it is still the most commonly used criteria for insignificant prostate cancer and hence included in this study [6].

The second analysis compared PC characteristics from prostatectomy specimens between men aged ≤55 years and those >55 years. This analysis is more robust in terms of actual pathological cancer differences but subject to selection bias as some men with low risk cancers may not have had curative treatment and older men may be more likely to have radiotherapy rather then surgery. Similarly, from the prostatectomy data subjects were identified as having insignificant prostate cancer as per the Stamey criteria [8] (tumour volume <0.5 cm^3^, no Gleason pattern 4 or 5, and no extracapsular spread) or high-risk prostate cancer as per the D’Amico risk grouping.

The statistics package SPSS version 21 was used to perform Chi-square analyses for categorical and dichotomous data, and Student’s t-tests for continuous data. Significance was set at *p* < 0.05. Ethics approval was sought and granted by the Western Sydney Local Health District Human Research Ethics Committee.

## Results

There were 794 prostate biopsy subjects identified for the study, representing a consecutive series. Of these, the DHM electronic database had complete information for a total of 598 prostate biopsy and 723 prostatectomy subjects, which were matched. Missing data was attributed to procedures being performed prior to routine electronic uploading of histopathology reports. There were no stored hard copies of these missing reports and hence they were excluded from the study.

Table 1 shows the demographic and clinicopathologic characteristics of the 598 subjects that underwent prostate biopsy. As demonstrated, the minority of subjects (15.6 %) were ≤55 years whilst 84.4 % were >55 years (*p* < 0.001). The most frequent pre-biopsy PSA value range was 4.1–10 for both men ≤55 years (59.1 %) and men > 55 (67.7 %) (*p* = 0.11). A Gleason score of 3 + 4 was the most common for both age groups (41.9 % ≤55 years and 45.7 % >55 years) (*p* = 0.50) as was a clinical stage of T1c (69.9 % ≤55 years and 67.5 % >55 years) (*p* = 0.65).

**Table 1:** Patient characteristics for prostate biopsy specimens for men ≤55 years compared to men >55 years

Characteristic	Age ≤55 years	Age >55 years	Total	Significance
Sample numbers	93 (15.6 %)	505 (84.4 %)	598 (100 %)	X^2^ = 283.9, *p* < 0.001
Age (years)				
-Median	53	64	63	
-Range	41–55	56–79	41–79	
-Mean	51.9	64.3	62.4	
Clinical stage				
cT1c	65 (69.9 %)	341 (67.5 %)	406 (67.9 %)	X^2^ = 0.20, *p* = 0.65
cT2a	10 (10.8 %)	64 (12.7 %)	74 (12.4 %)	X^2^ = 0.27, *p* = 0.61
cT2b	2 (2.2 %)	25 (5.0 %)	27 (4.5 %)	X^2^ = 1.43, *p* = 0.23
cT2c	14 (15.1 %)	73 (14.5 %)	87 (14.5 %)	X^2^ = 0.02, *p* = 0.88
cT3a	0 (0 %)	0 (0 %)	0 (0 %)	-
cT3b	0 (0 %)	1 (0.2 %)	1 (0.2 %)	X^2^ = 0.18, *p* = 0.67
Missing	2 (2.2 %)	1 (0.2 %)	3 (0.5 %)	X^2^ = 6.00, *p* = 0.01
PSA score (ng/mL)				
<3	9 (9.7 %)	43 (8.5 %)	52 (8.7 %)	X^2^ = 0.134, *p* = 0.72
3–4	12 (12.9 %)	41 (8.1 %)	53 (8.9 %)	X^2^ = 2.23, *p* = 0.14
4.1–10	55 (59.1 %)	342 (67.7 %)	397 (66.4 %)	X^2^ = 2.59, *p* = 0.11
10.1–20	14 (15.1 %)	73 (14.5 %)	87 (14.5 %)	X^2^ = 0.23, *p* = 0.88
>20.1	3 (3.2 %)	6 (1.2 %)	9 (1.5 %)	X^2^ = 2.2, *p* = 0.14
Gleason score				
3 + 3	32 (34.4 %)	135 (26.7 %)	167 (27.9 %)	X^2^ = 2.3, *p* = 0.13
3 + 4	39 (41.9 %)	231 (45.7 %)	270 (45.2 %)	X^2^ = 0.46, *p* = 0.50
4 + 3	13 (14.0 %)	69 (13.7 %)	82 (13.7 %)	X^2^ = 0.007, *p* = 0.94
8	5 (5.4 %)	29 (5.7 %)	34 (5.7 %)	X^2^ = 0.02, *p* = 0.89
9–10	4 (4.3 %)	41 (8.1 %)	45 (7.5 %)	X^2^ = 1.65, *p* = 0.20
Biopsy				
No. cores mean	14.3	13.9	13.9	t = 1.17, *p* = 0.24, Mean difference = 0.48, 95 % CI of difference = −0.33 to 1.30
% of positive cores mean	31	34	34	t = −1.51, *p* = 0.13, Mean difference = −3.73, 95 % CI of difference = −8.58 to 1.11

Table 2 illustrates the prostate biopsy outcomes by age. 14.0 % of men ≤55 years and 11.9 % >55 years had insignificant prostate cancer as per the Epstein criteria, with no significant difference between the two age groups (X^2^ = 0.32, df = 1, *p* = 0.57). Similarly, there was no significant difference between the two age groups in Gleason score >6 (X^2^ = 2.3, *p* = 0.13), Gleason score >7 (X^2^ = 1.2, *p* = 0.27), Gleason score as a continuous variable (t = −1.530, *p* = 0.13, mean difference = −0.195, 95 % CI of difference = −0.45 to 0.06), high-risk prostate cancer features (X^2^ = 0.007, *p* = 0.93) or pre-biopsy PSA scores (t = 0.977, *p* = 0.33, mean difference = 1.45, 95 % CI of difference = −1.50 to 4.41).

**Table 2:** Prostate biopsy outcomes for men ≤55 years compared to men >55 years

Factor	Age ≤55 years	Age >55 years	Total	Significance
Insignificant prostate cancer (Epstein criteria)	13 (14.0 %)	60 (11.9 %)	73 (12.2 %)	X^2^ = 0.32, *p* = 0.57
Total	93	505	598	
Gleason score				
>6	61 (65.6 %)	370 (73.3 %)	431 (72.1 %)	X^2^ = 2.3, *p* = 0.13
>7	9 (9.7 %)	70 (13.9 %)	79 (13.2 %)	X^2^ = 1.2, *p* = 0.27
Continuous variable	Mean = 2.03	Mean = 2.23		t = −1.53
	Std dev = 1.05	Std dev = 1.15		*p* = 0.13
				Mean difference = −0.20
				95 % CI of difference = −0.45 to 0.06
PSA comparison	Mean = 8.55	Mean = 7.10		t = 0.977
	Std dev = 14.26	Std dev = 3.98		*p* = 0.33
	Std error mean =1.48	Std error mean =0.18		Mean difference = 1.45
				95 % CI of difference = −1.50 to 4.41
High risk PC				
GS > 7	9 (12.5 %)	70 (13.9 %)	79 (13.2 %)	X^2^ = 1.2, *p* = 0.27
PSA > 20	3 (3.2 %)	6 (1.2 %)	9 (1.5 %)	X^2^ = 2.2, *p* = 0.14
Clinical stage > T2b	14 (15.1 %)	74 (14.7 %)	88 (14.7 %)	X^2^ = 0.03, *p* = 0.86
At least one feature of high risk PC	23 (24.7 %)	127 (25.1 %)	150 (25.1 %)	X^2^ = 0.007, *p* = 0.93

The patient characteristics of the 723 subjects that underwent prostatectomy were similar in nature to the prostate biopsy patient characteristics (Table 3). There were less men ≤55 years (15.2 %) than >55 years (84.8 %) (*p* < 0.001), a common pre-biopsy PSA value of 4.1–10 in both men ≤55 years (56.4 %) and >55 years (65.6 %) (*p* = 0.06),and a common Gleason score 3 + 4 (51.8 % in men ≤55 years, 53.2 % in men >55 years) (*p* = 0.79). Additionally, the pathological stage was most frequently T2 in men ≤55 years (65.5 %) and men >55 years (59.5 %) (*p* = 0.24). Most often there was no seminal vesicle involvement (92.7 % in men ≤55 years, 92.3 % in men >55 years) (*p* = 0.89) and negative lymph nodes (62.7 % in men ≤55 years, 62.6 % in men >55 years) (*p* = 0.99). When comparing PSA as a continuous variable for the prostatectomy subjects there was no significant difference between men ≤55 and men >50 years (t = −0.365, *p* = 0.72, mean difference = −0.29, 95 % CI of difference = −1.86 to 1.28).

**Table 3:** Prostatectomy patient characteristics and outcomes for men ≤55 years compared to men >55 years

Characteristic	Age ≤55 years	Age >55 years	Total	Significance
Sample numbers	110 (15.2 %)	613 (84.8 %)	723 (100 %)	X^2^ = 349.9, *p* < 0.001
Age (years)				
-Median	53	64	63	
-Range	41–55	56–79	41–79	
-Mean	52.0	64.2	62.4	
PSA score (ng/mL)				
<3	11 (10.0 %)	67 (10.9 %)	78 (10.8 %)	X^2^ = 0.08, *p* = 0.77
3–4	25 (22.7 %)	43 (7.0 %)	68 (9.4 %)	X^2^ = 27.02, *p* < 0.001
4.1–10	62 (56.4 %)	402 (65.6 %)	464 (64.2 %)	X^2^ = 3.45, *p* = 0.06
10.1–20	7 (6.4 %)	90 (14.7 %)	94 (13.4 %)	X^2^ = 5.56, *p* = 0.02
>20.1	4 (3.6 %)	8 (1.3 %)	12 (1.7 %)	X^2^ = 3.11, *p* = 0.08
Missing	1 (0.9 %)	3 (0.5 %)	4 (0.6 %)	X^2^ = 0.30, *p* = 0.59
Continuous variable	110	613	723	(t = −0.365, *p* = 0.72, mean difference = −0.29, 95 % CI of difference = −1.86 to 1.28)
Gleason score				
3 + 3	26 (23.6 %)	96 (15.7 %)	122 (16.9 %)	X^2^ = 4.23, *p* = 0.40
3 + 4	57 (51.8 %)	326 (53.2 %)	383 (53.0 %)	X^2^ = 0.70, *p* = 0.79
4 + 3	19 (17.3 %)	128 (20.9 %)	147 (20.3 %)	X^2^ = 0.75, *p* = 0.39
8	3 (2.7 %)	13 (2.1 %)	16 (2.2 %)	X^2^ = 0.16, *p* = 0.69
9–10	5 (4.5 %)	50 (8.2 %)	55 (7.6 %)	X^2^ = 1.73, *p* = 0.19
>6	84 (76.4 %)	517 (84.3 %)	601 (83.1 %)	X^2^ = 4.23, *p* = 0.40
>7	8 (7.3 %)	63 (10.3 %)	71 (9.8 %)	X^2^ = 0.95, *p* = 0.33
Continuous variable	mean = 2.13	mean = 2.34		t = −2.00
	Std dev = 0.96	Std dev = 1.04		*p* = 0.05
				Mean difference = −0.212
				95 % CI of difference = −0.42 to 0.00
Pathological stage				
pT2	72 (65.5 %)	365 (59.5 %)	437 (60.4 %)	X^2^ = 1.36, *p* = 0.24
pT3a	24 (21.8 %)	167 (27.2 %)	191 (26.4 %)	X^2^ = 1.41, *p* = 0.24
pT3b	14 (12.7 %)	80 (13.1 %)	94 (13.0 %)	X^2^ = 0.01, *p* = 0.93
pT4	0 (0 %)	0 (0 %)	0 (0 %)	-
Missing	0 (0 %)	1 (0.2 %)	1 (0.1 %)	X^2^ = 0.18, *p* = 0.67
Prostate weight	Mean = 46.40	Mean = 54.83		t = −5.11,
	Std dev = 14.86	Std dev = 20.72		df = 191.8,
				*p* < 0.001,
				95 % CI = −11.69 to −5.17
Tumour volume				
<0.5	21 (19.1 %)	109 (17.8 %)	130 (18.0 %)	X^2^ = 0.11, *p* = 0.74
0.5-1	23 (20.9 %)	107 (17.5 %)	130 (18.0 %)	X^2^ = 0.75, *p* = 0.39
1.1-2	17 (15.5 %)	106 (17.3 %)	123 (17.0 %)	X^2^ = 0.22, *p* = 0.64
2.1+	13 (11.8 %)	123 (20.1 %)	136 (18.8 %)	X^2^ = 4.15, *p* = 0.04
Missing	36 (32.7 %)	168 (27.4 %)	204 (28.2 %)	X^2^ = 1.30, *p* = 0.25
Mean tumour volume	Mean = 1.37	Mean = 1.82		t = −1.628,
	Std dev = 1.51	Std dev = 2.34		df = 516,
				*p* = 0.104,
				95 % CI = −1.01 to 0.95
Seminal vesicle				
Negative	102 (92.7 %)	566 (92.3 %)	668 (92.4 %)	X^2^ = 0.02, *p* = 0.89
Positive	8 (7.3 %)	47 (7.7 %)	55 (7.6 %)	
Lymph nodes				
Negative	69 (62.7 %)	384 (62.6 %)	453 (62.7 %)	X^2^ = 0.00, *p* = 0.99
Positive	4 (3.6 %)	16 (2.2 %)	20 (2.8 %)	X^2^ = 0.37, *p* = 0.55
Not taken	37 (33.6 %)	213 (29.5 %)	250 (34.6 %)	X^2^ = 0.05, *p* = 0.82
Insignificant prostate cancer (Stamey criteria)	10 (9.1 %)	40 (6.5 %)	50 (6.9 %)	X^2^ = 1.25, *p* = 0.26
Missing	10 (9.1 %)	29 (4.7 %)	39 (5.4 %)	
Total	110	613	723	
Clinical stage				
-Organ confined (pT2, N0NX)	72 (65.5 %)	363 (59.3 %)	435 (60.2 %)	X^2^ = 1.47, *p* = 0.23
-Non-Organ confined (pT3/4, N0-1)	38 (34.5 %)	249 (40.7 %)	287 (39.8 %)	
Margin positive	27 (24.5 %)	156 (25.4 %)	183 (25.3 %)	X^2^ = 0.40, *p* = 0.84
High risk PCa				
GS > 7	8 (7.3 %)	63 (10.3 %)	71 (9.8 %)	X^2^ = 0.95, *p* = 0.33
PSA > 20	4 (3.6 %)	8 (1.3 %)	12 (1.7 %)	X^2^ = 3.11, *p* = 0.08
Clinical stage > T2b	15 (13.6 %)	89 (14.5 %)	104 (14.4 %)	X^2^ = 0.06, *p* = 0.81
At least one feature of high risk PCa	22 (20.0 %)	147 (24.0 %)	169 (23.4 %)	X^2^ = 0.83, *p* = 0.36

The prostatectomy outcomes paralleled the prostate biopsy outcomes, with similar rates of insignificant prostate cancer as per the Stamey criteria (9.1 % in men ≤55 years, 6.5 % in men >55 years) and no statistically significant difference (X^2^ = 1.25, *p* = 0.26) (Table 3). Furthermore, there were similar mean tumour volumes (t = −1.63, df = 516, *p* = 0.10, 95 % CI = −1.01 to 0.95), Gleason score >6 (X^2^ = 4.23, *p* = 0.40), Gleason score >7 (X^2^ = 0.95, *p* = 0.33), Gleason score as a continuous variable (t = −2.000, *p* = 0.05, mean difference = −0.212, 95 % CI of difference = −0.42 to 0.00), margin positive status (X^2^ = 0.40, *p* = 0.84) and high-risk prostate cancer (X^2^ = 0.83, *p* = 0.36). As expected due to prostate hyperplasia with increasing age, the prostate weights in the men ≤55 years was significantly less than in men >55 years (t = −5.11, df = 191.8, *p* < 0.001, 95 % CI = −11.69 to −5.17). When pre-operative PSA levels are compared to prostatectomy tumour volumes, we find that PSA increases as tumour volume increases (t = 24.993, df = 515, *p* < 0.001). Also, men >55 years on average, have a higher PSA (6.83) and tumour volume (1.83) compared to men ≤55 years (mean PSA 6.22, mean tumour volume 1.37).

As described in Table 4, there were 27 men ≤50 years (4.5 %) and 571 men >50 years (95.5 %) who had prostate biopsies. Of these, 2 men ≤50 years (7.4 %) and 71 men >50 years (12.4 %) had insignificant prostate cancer (Epstein criteria), with no statistically significant difference between the two age groups (X^2^ = 0.55, *p* = 0.46). Within the prostatectomy subjects there were 32 men ≤50 years (4.4 %) and 691 men >50 years (95.6 %). Of these, 2 men ≤50 years (1.8 %) and 48 men >50 years (7.8 %) had insignificant prostate cancer (Stamey criteria) with no significant difference between the younger and older men (X^2^ = 0.008, *p* = 0.93) (Table 5).

**Table 4:** Prostate biopsy characteristics and outcomes for men ≤50 years compared to men >50 years

Characteristic	Age ≤50 years	Age >50 years	Total	Significance
Sample numbers	27 (4.5 %)	571 (95.5 %)	598 (100 %)	
Age (years)				
-Median	49	63	63	
-Range	41–50	51–79	41–79	
-Mean	47.9	63.0	62.4	
Insignificant prostate cancer (Epstein criteria)	2 (7.4 %)	71 (12.4 %)	73 (12.2 %)	X^2^ = 0.55, df = 1, *p* = 0.46
Total	27	571	598

**Table 5:** Prostatectomy characteristics and outcomes for men ≤50 years compared to men >50 years

Characteristic	Age ≤50 years	Age >50 years	Total	Significance
Sample numbers	32 (4.4 %)	691 (95.6 %)	723 (100 %)	
Age (years)				
-Median				
-Range	41–50	51–79	41–79	
-Mean				
Insignificant prostate cancer (Stamey criteria)	2 (1.8 %)	48 (7.8 %)	50 (6.9 %)	X^2^ = 0.008, df = 1, *p* = 0.93
Missing	3 (2.7 %)	36 (5.9 %)	39 (5.4 %)	
Total	110	613	723	

When the group of men ≤55 years was subdivided into men ≤50 and 51–55 years, similar rates of insignificant prostate cancer were found in both prostate biopsy and prostatectomy specimens. From the prostate biopsies, 2 men ≤50 years and 11 men 51–55 years had insignificant prostate cancer (X^2^ = 1.238, df = 1, *p* = 0.27). From the prostatectomy specimens, 2 men ≤50 years and 8 men 51–55 years had insignificant prostate cancer (X^2^ = 0.437, df = 1, *p* = 0.51).

## Discussion

Since the introduction of PSA testing, rates of prostate cancer diagnosis have dramatically increased whilst prostate cancer mortality has fallen [9]. Despite this, a large proportion of those diagnosed with prostate cancer will have insignificant prostate cancer [10] – many of whom will have unnecessary treatment and subsequently experience morbidity and mortality. It is therefore important to define a population that would optimally benefit from PSA testing.

We chose to analyse differences in prostate cancer pathology on biopsy specimens between the age groups as this avoids selection bias to some extent. There would be some selection bias in who gets a prostate biopsy with an abnormal PSA, however we would expect the bias would favour older men having more significant cancer as these men would be more likely to avoid/not be recommended a biopsy at lower PSA levels [11]. We also studied the differences in prostatectomy specimen pathology between the age groups because Epstein criteria has inherent inaccuracies in detecting insignificant cancers [6]. Prostatectomy specimens allow more accurate determination of pathology but are subject to more selection bias. Again, we would expect the bias would be toward older men having more significant cancers, as younger men would elect to have surgery for less significant cancers and opt for active surveillance less readily then older men as a result of different life expectancies and co-morbidities [12]. In Australia, PSA screening is performed by the primary care physicians and patients are generally referred to a urologist when the PSA is above the age specific reference range. We do not have data on the total number of men screened in each age group.

In our study population, there was no statistically significant difference in the rates of insignificant or high-risk prostate cancer between men ≤55 years and >55 years on either the prostate biopsy or prostatectomy specimens. Therefore, it would seem to follow that men ≤55 years would benefit from PSA testing by allowing early detection of significant prostate cancer in the same way as older men do. This in turn facilitates early treatment and prevents presentation with advanced disease. Early diagnosis of significant prostate cancer may be particularly important in men <55 years as high grade and locally advanced prostate cancer in this younger group may paradoxically have worse outcomes compared to their older counterparts - potentially reflecting differing prostate cancer biology in this population [13].

There is currently contradictory evidence in the literature about the benefit of prostate cancer screening in terms of reducing prostate cancer-specific mortality. The PLCO and ERSPC trials are often quoted as the most rigorous of the available studies, though these give conflicting results. PLCO concluded that there was no significant prostate cancer-specific mortality benefit (RR 1.15, 95 % CI 0.86 to 1.54) from screening, whilst ERSPC showed a significant benefit (RR 0.84, 95 % CI 0.73 to 0.95) [1]. Unfortunately, neither study included men <55 years, therefore do not reliably contribute to the discussion of PSA screening in this age group.

Of the trials that included men <55 years, the Goteborg population-based randomised trial showed a reduction in metastatic disease and prostate cancer-specific mortality when screening started at 50 years [14]. The Quebec trial included men as young as 45 years though has demonstrated no benefit in prostate-specific mortality with PSA testing when using an intention-to-treat analysis [15]. A Cochrane review exploring the prostate cancer-specific mortality according to the age of participants at screening identified no significant difference in prostate cancer-specific mortality regardless of whether the men were screened from age 45 years (RR 1.01; 95 % CI 0.76–1.33), 50 years (RR 0.93; 95 % CI 0.69–1.27) or 55 years (RR 1.12; 95 % CI 0.92–1.37) [1].

In a retrospective study, Twiss et al. (2005) found that men under 50 years had similar rates of significant prostate cancer as men over 50 years when analysing radical retropubic prostatectomy specimens - implying that there would be utility in PSA testing both populations [16]. In contrast, Howard et al. (2009) found that prostate cancer screening beginning at 40 years compared to 50 years would prevent less than 1 prostate cancer death per 1000 men screened [17]. This is due to the fact that the rate of prostate cancer detection in the younger age group is lower than older men. Our study has shown however that when a cancer is detected in this younger age group, it is no less aggressive.

The role of a baseline PSA test in younger men for predicting future risk of prostate cancer is also controversial. There is evidence that PSA testing in men in their 40’s can stratify risk of developing prostate cancer in the future, particularly if the baseline PSA is in the highest centiles above the median [18, 19]. This strategy would allow men with a high-risk PSA to undergo close monitoring, whilst sparing men with a low-risk PSA from regular PSA testing.

This raises the question of at which age PSA testing should actually be implemented. It may be when below a certain age there is a statistically significant difference in the rates of insignificant prostate cancer. In our data set, when the rates of insignificant prostate cancer in men ≤50 years were compared to those >50 years in both the prostate biopsy and prostatectomy specimens - there remained no difference, suggesting that screening may still be appropriate at this age.

We acknowledge that the definition of insignificant cancer has not been well defined clinically and validated and as such is controversial. Factors such as age and family history and life-expectancy will have to be included in any real pathological definition of insignificant cancer. In the absence of a well-established definition of insignificant cancer, we chose the Epstein criteria for needle biopsy and Stamey definition for prostatectomy specimens, as these are the most commonly reported.

Larger studies need to be designed to assess whether our findings remain consistent, particularly amongst different geographical/ethnic populations. Future trails should include men >40 years to more thoroughly assess prostate cancer screening benefits in terms of prostate cancer-specific mortality. Ideally, there would be exclusion of subjects with a family history of prostate cancer- as this is a known prostate cancer risk factor that alters PSA screening choice. Nevertheless, it is estimated that only 5–10 % of prostate cancer is related to a positive family history [20] and this would not explain the similarities in rates of high-risk prostate cancer in men ≤55 and >55 years in our study.

Limitations of this study include reliance on a third-party for availability of recorded and collected data. There were cases of missing subject data that resulted in the exclusion of some participants. Ideally, there would be an equal sample size of men ≤55 and >55 years in the study, however if consecutive subjects are chosen this is challenging to accomplish as more men >55 years have PSA testing and prostate biopsies. Screening calculators such as the Prostate Cancer Prevention Trial (PCPT) screening calculator may be used for an individualised assessment of prostate cancer risk.

## Conclusions

PSA testing remains controversial and guidelines will continue to evolve in the coming years. We found no significant difference in the rates of insignificant and high-risk prostate cancer between men ≤55 years and >55 years, in either the prostate biopsies or prostatectomy specimens. The optimal age to begin PSA testing is yet unclear, however, our findings suggest that it should be less than 55 years. Further trials need to be performed with comparable sample sizes and controlling of risk factors to assess the utility of PSA screening in younger men.

## Abbreviations

PC: Prostate cancer
PSA: Prostate specific antigen
AUA: American Urological Association
DRE: Digital rectal examination

## References

[1] Ilic D, Neuberger MM, Djulbegovic M, Dahm P (2013). Screening for prostate cancer. Cochrane Database Syst Rev.

[2] Carter HB, Albertsen PC, Barry MJ (2013). Early detection of prostate cancer: AUA Guideline. J Urol.

[3] Ploussard G, Epstein JI, Montironi R (2011). The contemporary concept of significant versus insignificant prostate cancer. Eur Urol.

[4] Schroder FH, Hugosson J, Roobol MJ (2014). Screening and prostate cancer mortality: results of the European Randomised Study of Screening for Prostate Cancer (ERSPC) at 13 years of follow-up. Lancet.

[5] Andriole GL, Crawford ED, Grubb RL (2012). Prostate cancer screening in the randomized Prostate, Lung, Colorectal, and Ovarian Cancer Screening Trial: mortality results after 13 years of follow-up. J Natl Cancer Inst.

[6] Oon SF, Watson RW, O'Leary JJ, Fitzpatrick JM (2011). Epstein criteria for insignificant prostate cancer. BJU Int.

[7] Hernandez DJ, Nielsen ME, Han M, Partin AW (2007). Contemporary evaluation of the D'amico risk classification of prostate cancer. Urology.

[8] Stamey TA, Freiha FS, McNeal JE, Redwine EA, Whittemore AS, Schmid HP (1993). Localized prostate cancer. Relationship of tumor volume to clinical significance for treatment of prostate cancer. Cancer.

[9] Oliver SE, May MT, Gunnell D (2001). International trends in prostate-cancer mortality in the “PSA ERA”. Int J Cancer.

[10] Haas GP, Delongchamps N, Brawley OW, Wang CY, de la Roza G (2008). The worldwide epidemiology of prostate cancer: perspectives from autopsy studies. Can J Urol.

[11] Oesterling JE, Jacobsen SJ, Chute CG (1993). Serum prostate-specific antigen in a community-based population of healthy men. Establishment of age-specific reference ranges. JAMA.

[12] Stangelberger A, Waldert M, Djavan B (2008). Prostate cancer in elderly men. Rev Urol.

[13] Lin DW, Porter M, Montgomery B (2009). Treatment and survival outcomes in young men diagnosed with prostate cancer: a Population-based Cohort Study. Cancer.

[14] Hugosson J, Carlsson S, Aus G (2010). Mortality results from the Goteborg randomised population-based prostate-cancer screening trial. Lancet Oncol.

[15] Labrie F, Candas B, Cusan L (2004). Screening decreases prostate cancer mortality: 11-year follow-up of the 1988 Quebec prospective randomized controlled trial. Prostate.

[16] Twiss C, Slova D, Lepor H (2005). Outcomes for men younger than 50 years undergoing radical prostatectomy. Urology.

[17] Howard K, Barratt A, Mann GJ, Patel MI (2009). A model of prostate-specific antigen screening outcomes for low- to high-risk men: information to support informed choices. Arch Intern Med.

[18] Vickers AJ, Ulmert D, Sjoberg DD (2013). Strategy for detection of prostate cancer based on relation between prostate specific antigen at age 40–55 and long term risk of metastasis: case-control study. BMJ.

[19] Lilja H, Cronin AM, Dahlin A (2011). Prediction of significant prostate cancer diagnosed 20 to 30 years later with a single measure of prostate-specific antigen at or before age 50. Cancer.

[20] Hemminki K, Czene K (2002). Age specific and attributable risks of familial prostate carcinoma from the family-cancer database. Cancer.

